# Effects of vessel traffic and ocean noise on gray whale stress hormones

**DOI:** 10.1038/s41598-022-14510-5

**Published:** 2022-11-03

**Authors:** Leila S. Lemos, Joseph H. Haxel, Amy Olsen, Jonathan D. Burnett, Angela Smith, Todd E. Chandler, Sharon L. Nieukirk, Shawn E. Larson, Kathleen E. Hunt, Leigh G. Torres

**Affiliations:** 1 Geospatial Ecology of Marine Megafauna Lab, Department of Fisheries, Wildlife, and Conservation Science, Marine Mammal Institute, Oregon State University, 2030 SE Marine Science Dr, Newport, OR 97365 USA; 2 Institute of Environment, College of Arts, Science & Education, Florida International University, 3000 NE 151st St, North Miami, FL 33181 USA; 3 Pacific Northwest National Laboratory, 1529 W Sequim Bay Rd, Sequim, WA 98382 USA; 4 Cooperative Institute for Marine Resources Studies, Oregon State University, 2030 SE Marine Science Dr, Newport, OR 97365 USA; 5 Conservation Programs and Partnerships, Seattle Aquarium, 1483 Alaskan Way Pier 59, Seattle, WA 98101 USA; 6 Aerial Information Systems Laboratory, Forest Engineering, Resources and Management Department, Oregon State University, Oregon, USA; 7 Department of Biology, Smithsonian-Mason School of Conservation, George Mason University, Fairfax, VA USA

**Keywords:** Endocrinology, Animal physiology

## Abstract

Human use of marinescapes is rapidly increasing, especially in populated nearshore regions where recreational vessel traffic can be dense. Marine animals can have a physiological response to such elevated human activity that can impact individual health and population dynamics. To understand the physiological impacts of vessel traffic on baleen whales, we investigated the adrenal stress response of gray whales (*Eschrichtius robustus*) to variable vessel traffic levels through an assessment of fecal glucocorticoid metabolite (fGC) concentrations. This analysis was conducted at the individual level, at multiple temporal scales (1–7 days), and accounted for factors that may confound fGC: sex, age, nutritional status, and reproductive state. Data were collected in Oregon, USA, from June to October of 2016–2018. Results indicate significant correlations between fGC, month, and vessel counts from the day prior to fecal sample collection. Furthermore, we show a significant positive correlation between vessel traffic and underwater ambient noise levels, which indicates that noise produced by vessel traffic may be a causal factor for the increased fGC. This study increases knowledge of gray whale physiological response to vessel traffic and may inform management decisions regarding regulations of vessel traffic activities and thresholds near critical whale habitats.

## Introduction

Marine ecosystems have experienced a dramatic rise in commercial and recreational shipping and vessel traffic^1–3^. These forms of transport can be associated with negative impacts on marine wildlife populations^4,5^, increased risk of mortality to cetaceans through ship strikes^6,7^, and increased noise levels in the marine environments^8–10^. Baleen whales are particularly vulnerable to acoustic disturbance caused by shipping and vessel traffic as it overlaps with the frequencies that are primarily used for communication, navigation and foraging^11,12^. Such frequency overlap can cause “acoustic masking” whereby transmission or reception of acoustic cues is degraded due to elevated ambient noise, which may potentially hinder a cetacean’s ability to perform important life history activities^11^. Therefore, elevated recurrence of both lethal and non-lethal interactions between vessel traffic and cetaceans may impact individual health and population viability.

Human-wildlife interactions can cause negative outcomes at short and long-term scales, yet longitudinal monitoring of impacts of human activities on long-lived wildlife populations can be difficult, often requiring considerable resources and contextual individual-level information (i.e., demographic data)^13^. Thus, studies have primarily focused on short-term behavioral responses to directly associate the cause (i.e., disturbance source) to the outcome (i.e., behavioral response)^13,14^. Multiple short-term impacts of vessel encounters on marine mammals have been documented, including altered behavioral states^15–18^, increased group cohesion^13,19,20^, and displacement^15,21^. Additionally, individuals may recognize vessels as a risk, and thus avoid them by employing other anti-predatory tactics^17–19,21^. However, many factors can influence the intensity of a response, including vessel movements^19,21^, distance to the vessel^22^, and number of vessels^23^, as well as the social context, quality of prey patches, health condition and history of previous vessel encounters by an individual^24^. Such factors may even encourage individuals to tolerate disturbances instead of escaping^25^. In this context, it is important to highlight that the absence of short-term behavioral shifts does not imply the absence of effects caused by vessel interactions or other stressors^13,26^, and highlights the need for studies on the physiological impacts of human disturbance to cetaceans, particularly across longer periods to help address potential consequences to population dynamics^27,28^.

Observations of individual physiological responses to disturbance can identify important potential shifts in homeostatic state. For example, vessel encounters have been correlated with the metabolic rates of bottlenose dolphins (*Tursiops truncatus*)^21^ and humpback whales (*Megaptera novaeangliae*)^29^ as determined through respiration rates and surfacing interval. Variations in stress-related hormone (e.g., glucocorticoids [GCs]: cortisol, corticosterone) concentrations relative to vessel abundance and related shifts in ambient noise have also been reported for killer whales (*Orcinus orca*)^30^ and North Atlantic right whales (NARWs; *Eubalaena glacialis*)^31^. Although these few studies on the physiological responses of marine mammals to vessel traffic have been conducted, assessments remain scarce. Baleen whale physiology, in particular, is one of the most poorly understood of all mammals given inherent difficulties in sample collection^32,33^. Nevertheless, new techniques enable noninvasive or minimally invasive methods to collect samples, including feces, respiratory vapor, and blubber^31–35^, which have proven to be reliable techniques to monitor baleen whale physiology.

In fact, fecal samples have been successfully collected from various baleen whale species, including NARWs^33,36^, humpback whales^37,38^, blue whales (*Balaenoptera musculus*)^39,40^, and gray whales (*Eschrichtius robustus*)^41,42^ to assess concentrations of steroid hormones (e.g., GCs, androgens, progestogens, mineralocorticoids)^33,41^ that are eventually cleared from blood circulation by the liver and excreted via the urine and feces^43,44^. The GC metabolites in feces tend to increase in concentration hours or days following a stress exposure, with the time lag determined by species-specific gut passage time^43,44^. Thus, fecal GCs (fGC) can be used to monitor physiological responses to acute disturbances provided the appropriate time lag to excretion can be determined (often 1 to 2 days in large mammals^44^).

Multiple factors can confound the assessment of hormone concentrations in feces^42,45,46^, such as sex, age, nutritional status, reproductive state, and environmental factors (e.g., temperature^47^), all of which should be accounted for at an individual level to reliably relate whale GCs to a specific anthropogenic disturbance. Hence, this study aimed to better understand how individual baleen whales physiologically respond to vessel traffic while controlling for multiple confounding factors. Furthermore, we investigate correlations between vessel traffic and ocean noise levels to add explanatory power to the root cause of elevated vessel traffic effects on fGC concentrations in gray whales. Our research focuses on a sub-group of the Eastern North Pacific (ENP) gray whale population called the Pacific Coast Feeding Group (PCFG), that forages along the Oregon coast, USA, between June and November^48^. In this study area, PCFG gray whales are exposed to varying degrees and sources of both vessel traffic^15^ and ocean noise^49,50^.

Our objectives are to (1) describe the relationship between vessel counts leaving the ports each day with Sound Pressure Levels (SPL) recorded by a hydrophone outside the port entrance, (2) determine the effects of vessel traffic and potential confounding variables (i.e., body condition, age, sex, time) on gray whale fGC concentrations, and (3) determine the time lag between vessel traffic and fGC concentration responses in gray whales, which likely represents the gut passage time. We hypothesize a correlation between vessel traffic and ocean noise levels and predict that both vessel traffic and ocean noise levels will be positively correlated with gray whale fGC concentrations. Given the data deficiency regarding physiological impacts of vessel traffic and resulting ocean noise on baleen whales, this study addresses a critical knowledge gap that may inform vessel traffic and acoustic disturbance policies (e.g., number of vessels, speed, proximity) to reduce impacts on individual physiology and population dynamics of baleen whales.

## Results

**Figure 1:**
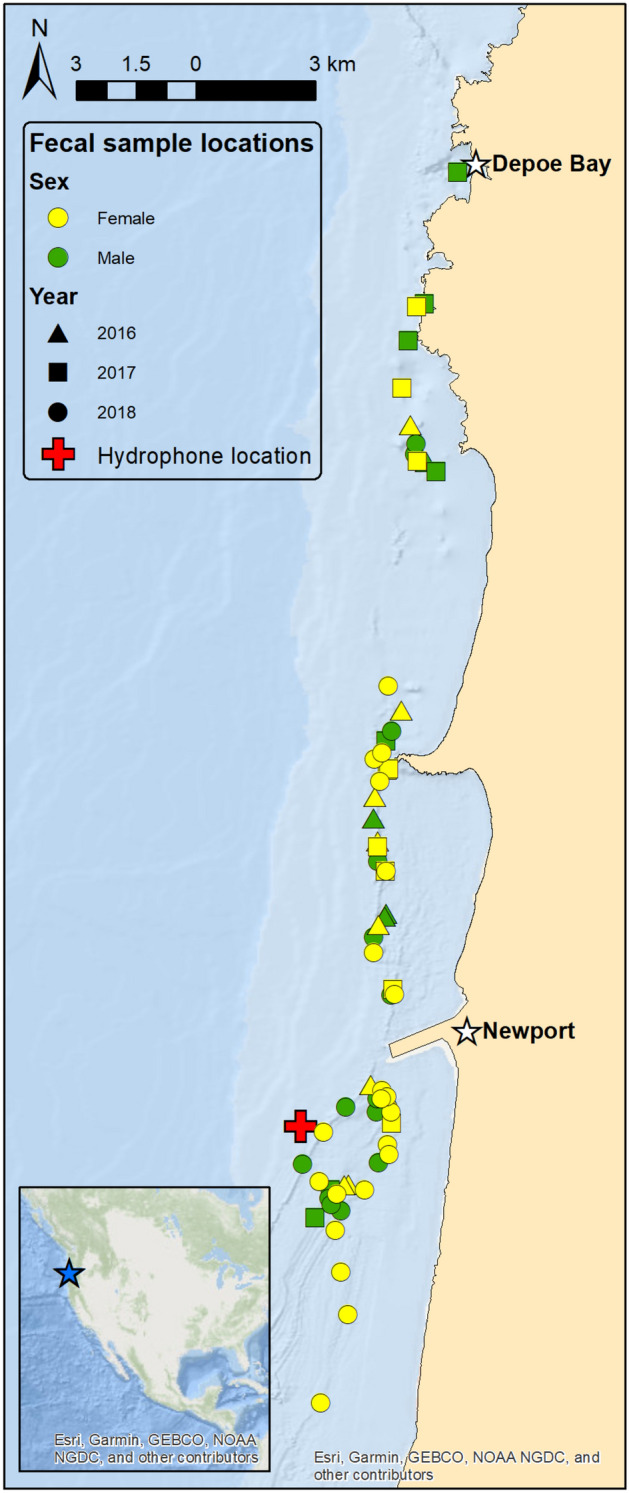
Location of field site off the central Oregon, USA coast (blue star on inset map), hydrophone deployment site (red cross), and gray whale fecal sample collections symbolized by color and shape to represent sex and year. The ports of Newport and Depoe Bay are represented by white stars. The Newport white star is also representing the anemometer station (NPOW3) from which the wind speed data were retrieved from. Figure created in ArcGIS software (version 10.8).

Data were collected during three gray whale foraging seasons (May to October of 2016–2018) along the central coast of Oregon, USA. We analyzed 411 days of vessel count data during the 2016–2018 field seasons, and 1128 hours of acoustic recordings from a total of 235 days during the 2017–2018 seasons. Furthermore, a total of 67 gray whale fecal samples were collected from 15 different mature males (n = 27) and 14 mature females (n = 40) (Fig. 1; Appendix S1: Table S1; Fig. S1). Fourteen fecal sample concentrations were below the limit of detection (< LOD) for fGC, four for progestin metabolites (fP), and ten for androgen metabolites (fA), while 19 fecal samples had no corresponding Body Area Index (BAI; a metric of body condition derived from drone photogrammetry) information. Therefore, these samples had missing values (NA) in the linear mixed models (LMM) analyses for the respective variables. In total, we had variable sample sizes by year and sex (males: 4 in 2016, 7 in 2017, and 16 in 2018; females: 9 in 2016, 9 in 2017, and 22 in 2018). If we only consider the samples with detectable fGC concentrations, the sample size is reduced to 0 in 2016, 5 in 2017, and 13 in 2018 for males, and 6 in 2016, 9 in 2017, and 20 in 2018 for females (N = 53).

Photo-identification and sighting history analysis of the 29 gray whales that the 67 fecal samples came from and included in this study indicated that the mean number of days these whales were resident in the study area prior to sampling was 32.96 (SD = 39.57, min = 0, max = 120). Despite the large SD and that 20 of these individuals were not observed in our study system prior to the day of sample collection (min = 0 days), an assessment of the total number of days these sampled whales were observed in our study region across the entire sampling year also indicates high residency time (mean = 59.07, SD = 42.51, min = 1, max = 125). It is important to highlight that (1) a whale could have been in the study area before first recorded by our team, and (2) a whale may have left the study area after we first observed it in a year and then returned. Overall, these sighting results demonstrate high residency time of sampled whales within the study area and are therefore likely exposed to the noise, wind and vessel data assessed in this study.

**Figure 2:**
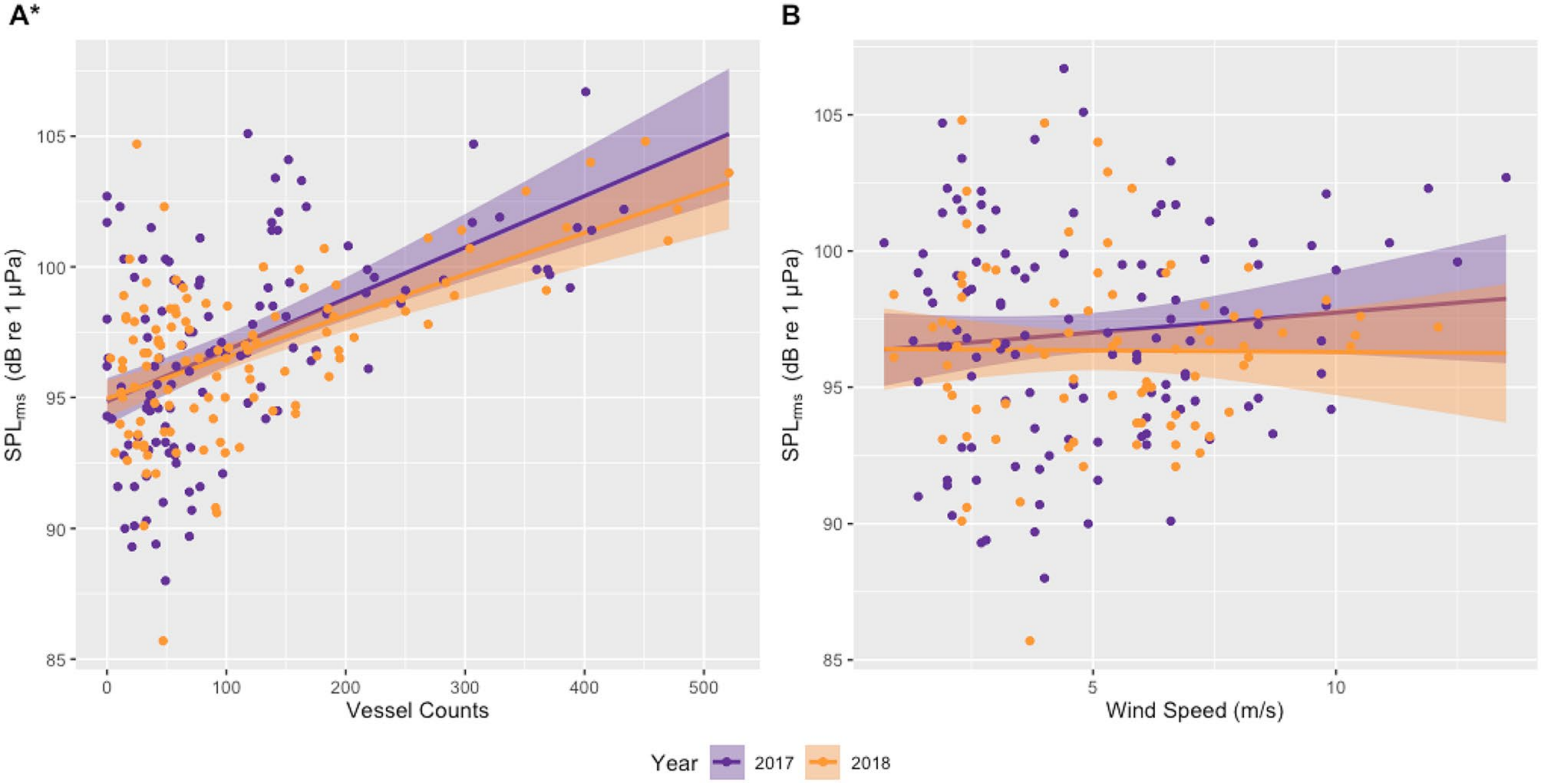
Linear correlations between noise levels (daily median root mean square [rms] sound pressure level [SPL] in dB [re 1 μPa]; 50–1000 Hz) recorded on a hydrophone deployed outside the Newport harbor entrance during June to October of 2017 and 2018 and (**A**) vessel counts in Newport and Depoe Bay, Oregon, USA, and (**B**) daily median wind speed (m/s) from an anemometer station located on South Beach, Newport, Oregon, USA (station NWPO3). Asterisk indicates significant correlations between SPL and vessel counts in both years.

Daily counts of unique vessels from the two ports in the study area (Newport and Depoe Bay) were highly variable, ranging from 0 to 521 vessels counted per day, with higher mean numbers of vessels in July (mean = 135) and August (mean = 133). Underwater noise levels were positively correlated with vessel counts (2017: rate of change = 0.020, F_1,114_ = 43.72, R^2^ = 0.271, *p* < 0.001; 2018: rate of change = 0.016, F_1,112_ = 54.92, R^2^ = 0.323, *p* < 0.001; Fig. 2A). Most whales were sampled within 10 km of the hydrophone (44.62% < 5 km; 15.38% between 5 and 10 km; 40% > 10 km), indicating likely exposure to the vessel traffic noise recorded.

**Figure 3:**
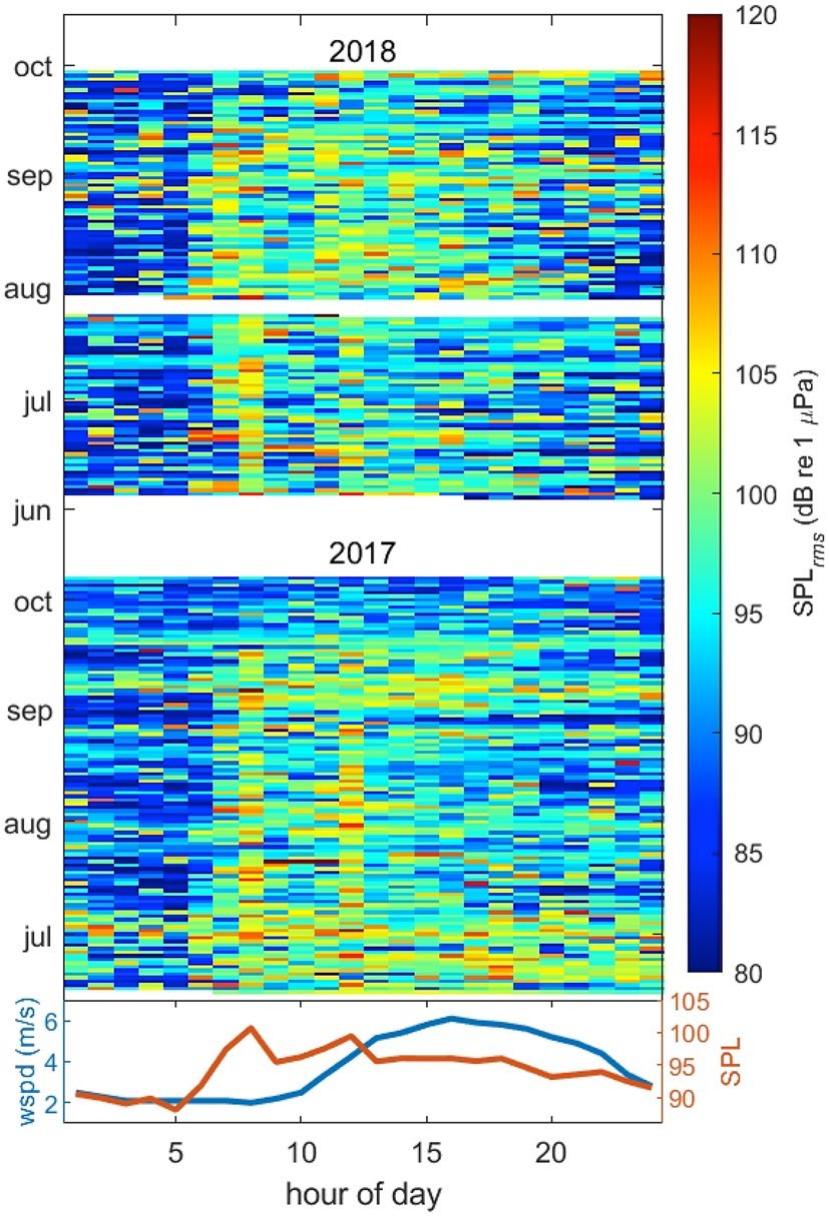
Median noise levels (root mean square sound pressure levels—SPL_rms_) for each hour of each day recorded on a hydrophone (50–10,000 Hz) deployed outside the Newport harbor entrance during June to October of 2017 (middle plot) and 2018 (upper plot), and hourly median noise level (SPL) against hourly median wind speed (lower plot) from an anemometer station located on South Beach, Newport, Oregon, USA (station NWPO3) over the same time period.

Daily median wind speed also fluctuated, ranging from 0.7 to 13.5 m/s, with higher median wind speeds occurring in July (5.95 m/s) and August (5.90 m/s). No correlation between wind speed and noise levels was observed in 2017 (rate of change = 0.146, F_1,114_ = 1.201, R^2^ = 0.002, *p* = 0.275) or 2018 (rate of change =  − 0.012, F_1,81_ = 0.006, R^2^ =  − 0.012, *p* = 0.937; Fig. 2B). Visual assessment of the temporal patterns in ambient sound level variation during the study periods (Fig. 3) indicates a strong, sharp onset maximum in underwater noise between 6 and 8 a.m. most days, which is coincident with peak recreational and sportfishing charter vessel traffic as boats transit out of the harbor to fishing grounds. A smaller peak in noise levels occurs at noon, which may represent a significant number of half-day fishing charters returning to the harbor around the same time. The absence of other similar peaks in noise levels during the day likely relates to the variable return times of vessels based on differences in fishing success (e.g., catch limits) or rising weather conditions and return distances and vessel speeds from the fishing grounds. Wind speeds in this study system typically begin to rise after 10 a.m. and peak between 3 and 4 p.m., which is an incongruous pattern with recorded ambient noise levels and further evidence that noise levels are dominated by vessel activity. Between midnight and 5 a.m., a steady decline of sound pressure levels to their lowest values is also noted as vessel traffic and wind speeds also reach their daily minimum.

**Figure 4:**
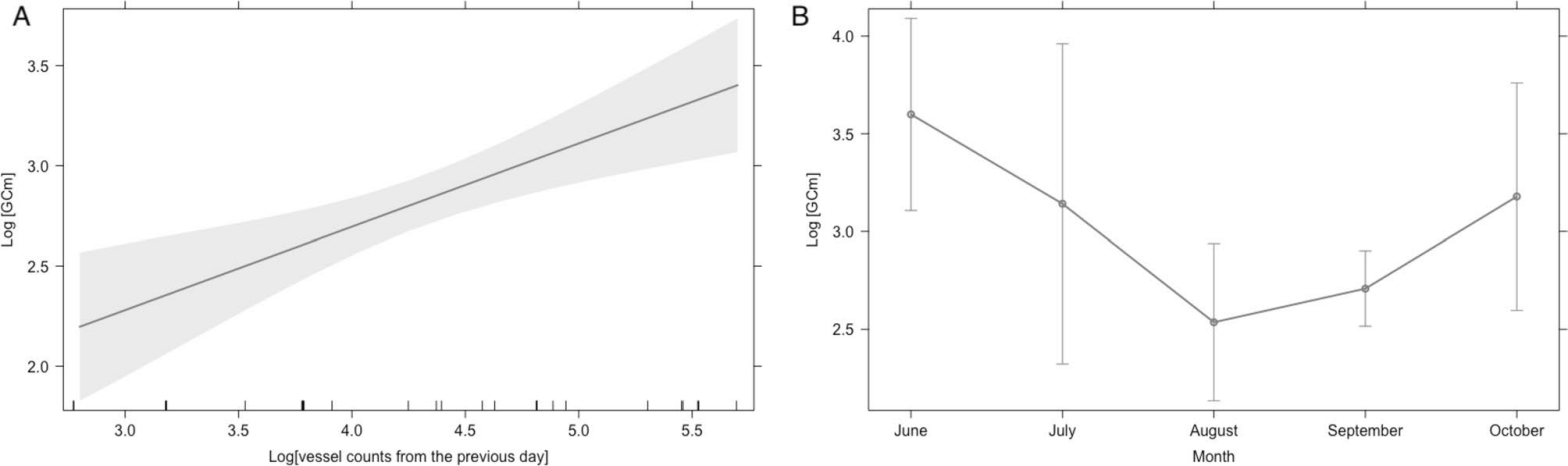
(**A**) The effect of vessel counts in Newport and Depoe Bay (Oregon, USA) on the day previous to fecal sample collection on gray whale fecal glucocorticoid metabolite (fGC) concentrations, derived from linear mixed model results with whale identification as random effect (Table 2; Model 2). (**B**) The effect of month on the gray whale fecal glucocorticoid metabolite (fGC) concentrations, also derived from linear mixed model results with whale identification as random effect (Table 2; Model 2). Data collected between May and October of 2016, 2017 and 2018. Asterisk indicates significant correlations between the variables in both A and B. Note difference in axes scales between A and B.

**Figure 5:**
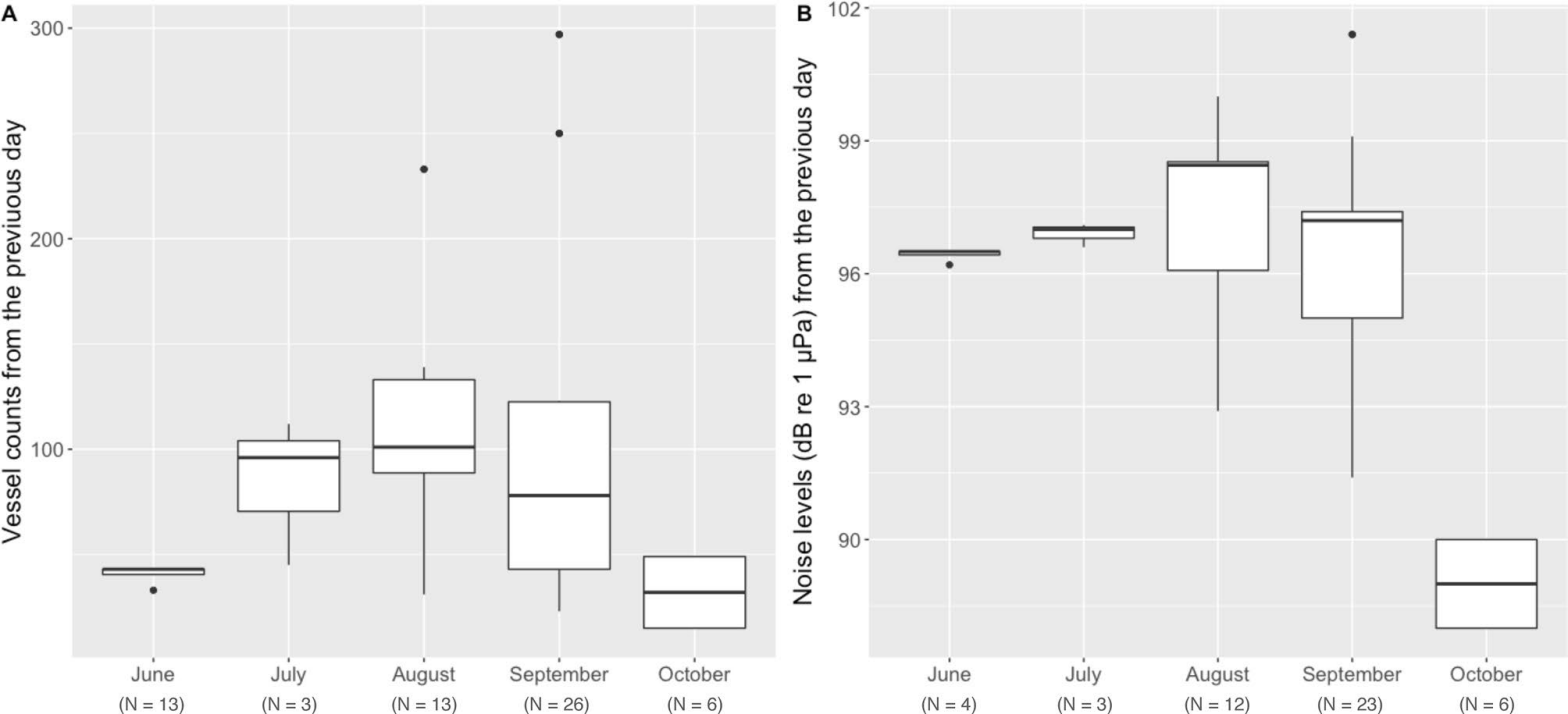
Boxplots displaying the variation in (**A**) vessel counts in Newport and Depoe Bay (Oregon, USA) on the day previous to fecal sample collection on gray whale fecal glucocorticoid metabolite (fGC), and (**B**) noise levels (daily median SPLrms, in dB [re 1 μPa]; 50 Hz - 1,000 Hz) recorded on a hydrophone deployed outside the Newport harbor entrance on the day previous to fecal sample collection summarized by month during 2017 and 2018. The boxplot upper and lower limits represent the 25% and 75% quantiles, the mid-lines indicate the medians, the whiskers represent minimum and maximum values (range), and the dots indicate outliers.

LMM analyses identified model 2 as the best model based on the lowest AIC. Model 2 included vessel counts from the previous day, sex, month, year, BAI, and fP and fA concentrations (Table 1, model 2; AIC = 58.55, df = 14, R^2^m = 0.64, R^2^c = 0.64), with vessel counts from previous day (F_1,17_ = 14.88, p = 0.001; Fig. 4A) and month (F_1,17_ = 4.12, p = 0.016) both significant (Table 2). Once the most influential temporal scale of vessel counts on fGC was determined (e.g., day before the sample collection), 17 additional LMMs were run with varied combinations of the fixed effects (Table 1, A–Q), but model 2 still displayed the lowest AIC. The months of August and September were significantly different from June in the model (Table 2). Although a linear regression between fGC concentrations and vessel counts of previous day, partitioned by month, found no significant correlation (p > 0.05), the plot (Fig. 4B) indicates that fGC was lower during the two months of August and September compared to June. Our posthoc analyses indicated no significant differences in vessel counts (ANOVA: F_(4,56)_ = 1.701, p = 0.163; Fig. 5A) and significant differences in ambient noise levels (ANOVA: F_(4,43)_ = 14.32, p < 0.001; Fig. 5B) on the previous day of fecal sample collection when partitioned by month. Yet, both vessel counts and ambient noise levels on the previous day of fecal sample collection display the same trend, with increased values in the months of August and September, and reduced values in the month of October.

**Table 1:** Linear mixed model (LMM) selection parameters of gray whale fecal glucocorticoid metabolite (fGC) concentrations relative to vessel counts, sex, year, progestin metabolites (fP), androgen metabolites (fA) and body condition (Body Area Index: BAI).

Models	DF	AIC	R^2^m	R^2^c
0) fGC ~ sex + month + year + fP + fA + BAI + (1|whale ID)	14	67.81511	0.45	0.49
1) fGC ~ vessels_sameday_ + sex + month + year + fP + fA + BAI + (1|whale ID)	14	64.21275	0.54	0.54
**2) fGC ~ vessels**_**prev1**_** + sex + month** + **year + fP + fA + BAI + (1|whale ID)**	**14**	**58.5528**	**0.64**	**0.64**
3) fGC ~ vessels_prev2sum_ + sex + month + year + fP + fA + BAI + (1|whale ID)	14	59.86415	0.61	0.61
4) fGC ~ vessels_prev3sum_ + sex + month + year + fP + fA + BAI + (1|whale ID)	14	64.81003	0.5	0.5
5) fGC ~ vessels_prev4sum_ + sex + month + year + fP + fA + BAI + (1|whale ID)	14	68.19622	0.41	0.43
6) fGC ~ vessels_prev5sum_ + sex + month + year + fP + fA + BAI + (1|whale ID)	14	67.24901	0.45	0.56
7) fGC ~ vessels_prev6sum_ + sex + month + year + fP + fA + BAI + (1|whale ID)	14	68.38497	0.4	0.4
8) fGC ~ vessels_prev7sum_ + sex + month + year + fP + fA + BAI + (1|whale ID)	14	68.10813	0.4	0.4
A) fGC ~ vessels_prev1_ + sex + month + year + fP + fA + (1|whale ID)	13	91.82038	0.28	0.3
B) fGC ~ vessels_prev1_ + sex + month + year + fP + (1|whale ID)	12	103.6393	0.2	0.2
C) fGC ~ vessels_prev1_ + sex + month + year + (1|whale ID)	11	110.6568	0.1	0.1
D) fGC ~ vessels_prev1_ + sex + month + (1|whale ID)	9	106.5958	0.08	0.08
E) fGC ~ vessels_prev1_ + sex + (1|whale ID)	5	97.52082	0.05	0.05
F) fGC ~ vessels_prev1_ + month + (1|whale ID)	8	104.8685	0.04	0.04
G) fGC ~ vessels_prev1_ + year + (1|whale ID)	6	100.0222	0.05	0.05
H) fGC ~ vessels_prev1_ + fP + (1|whale ID)	5	88.37007	0.13	0.13
I) fGC ~ vessels_prev1_ + fA + (1|whale ID)	5	84.47714	0.09	0.09
J) fGC ~ vessels_prev1_ + (1|whale ID)	4	95.57796	0.01	0.01
K) fGC ~ vessels_prev1_ + sex + month + year + fP + BAI + (1|whale ID)	13	67.12452	0.54	0.68
L) fGC ~ vessels_prev1_ + sex + month + year + fA + BAI + (1|whale ID)	13	60.16235	0.61	0.61
M) fGC ~ vessels_prev1_ + sex + month + year + BAI + (1|whale ID)	12	71.2999	0.48	0.48
N) fGC ~ vessels_prev1_ + sex + month + BAI + (1|whale ID)	10	67.72778	0.48	0.52
O) fGC ~ vessels_prev1_ + sex + BAI + (1|whale ID)	6	65.35813	0.37	0.37
P) fGC ~ vessels_prev1_ + month + BAI + (1|whale ID)	9	64.00439	0.49	0.51
Q) fGC ~ vessels_prev1_ + BAI + (1|whale ID)	5	61.61131	0.37	0.37

All models used whale identification (whale ID) as a random factor to account for pseudoreplication. Models 1–8 included the same predictor variables except vessel counts, which was assessed at different temporal scales in each model and ranged from the count of vessels on the same day of the fecal sample collection (vessels_sameday_, model 1) to the count of vessel within the seven previous days (vessels_prev7sum_, model 8). Model zero is the null model, which does not include vessels. Once the most influential temporal scale for vessel counts was determined (i.e., previous day; Model 2), 17 additional LMMs were run with varied combinations of the fixed effects (stepwise process, models A-Q; below the thin black line). Model in bold represents the selected model based on the lowest Akaike Information Criterion (AIC). The degrees of freedom for each model are indicated by DF. The fit of each model is represented by the marginal R^2^ (R^2^m; variance explained by fixed effects) and the conditional R^2^ (R^2^c; variance explained by both fixed and random effects).

**Table 2:** Parameter estimates from the chosen generalized linear mixed model (model 2) for gray whale fecal glucocorticoid metabolite concentrations using vessel counts from the previous day, sex, month, year, progestin metabolites, androgen metabolites, and body condition (Body Area Index: BAI) as fixed effects and whale identification as random factor.

Random effects	Variance	Standard deviation	Fixed effects	Estimate	Standard error	*p* value
Whale ID	0.000	0.000	Vessel counts from the previous day	0.415	0.108	0.001*
Residual	0.099	0.315	Sex	− 0.270	0.179	0.150
			Year (2017)	0.527	0.361	0.163
			Year (2018)	0.577	0.372	0.140
			Progestin metabolites	0.170	0.122	0.182
			Androgen metabolites	− 0.044	0.065	0.508
			Body Area Index	− 0.027	0.043	0.541
			Month (July)	− 0.457	0.482	0.356
			Month (August)	− 1.063	0.278	0.001*
			Month (September)	− 0.890	0.272	0.004*
			Month (October)	− 0.421	0.348	0.244

*Significance (*p* < 0.05).

## Discussion

This study demonstrates that increased vessel activity is a contributing factor in the stress physiology of gray whales. This finding is significant due to increasing human activities in oceans worldwide and should be incorporated into decision making by environmental managers when considering mitigation of impacts on baleen whale populations. Although the driver of this physiological response to vessel traffic remains undetermined, we hypothesize that elevated ocean noise from increased vessel traffic may be a causative factor of increased fGC concentrations. This hypothesis is based on a strong positive correlation between vessel counts from nearby ports and variable ambient noise levels (Fig. 2A), and between fGC concentrations and increased vessel counts (LMM; Table 2) from the previous day. Our assessment of the relationship between whale stress, vessel counts, and ambient noise occurred at varied temporal scales and accounted for confounding factors potentially influencing ambient sound levels (i.e., wind) and whale fGC concentrations, (i.e., body condition as an indicator of nutritional state, year, sex, age, and reproductive hormone concentrations). The amount of vessel traffic, and not wind speed, was correlated with underwater sound levels (50–1000 Hz) in our nearshore study area, which agrees with previous studies showing vessel traffic as the primary contributor to ocean noise in shallow coastal regions with significant fishing, recreation and tourism activities^51–54^. Our findings of a physiological response of a cetacean to elevated vessel traffic, with correlations to synoptic ocean noise levels, is important information for inclusion in the Population Consequences of Disturbance (PCoD)^27^ and Population Consequences of Multiple Stressors (PCoMS)^28^ frameworks to estimate impacts of disturbance events on population dynamics.

Our work highlights the utility of combining information on internal drivers (i.e., BAI, reproductive hormones) with external drivers (i.e., vessel counts, year) of mammalian “stress response”, since all these variables are known to affect physiological responses to acute stressors. Given the significant correlation between fGC and BAI in gray whales^42^, it was particularly important to include this nutritional index in our assessment of stress response. By incorporating multiple factors potentially impacting animal physiology into a single analysis, the unexplained variability of fecal hormone data can be better resolved to provide a clearer understanding of the major factors that affect stress responses in a field setting. While many questions remain regarding the drivers, timing, and repercussions of variable baleen whale hormone concentrations, we have demonstrated the feasibility of these methods and the correlation between vessel traffic and gray whale stress response.

Our analysis detected a statistically significant difference in fGC response by month (model 2; Table 2). The fGC response in gray whales was significantly lower in the months of August and September relative to June (Fig. 4B), which might be related to improved whale nutritive condition and/or prey availability during the later months of the foraging season. This hypothesis is supported by previous studies that determined increased body condition of gray whales in the later months of their foraging season^55^ and a correlation between body condition and fGC^42^. The increased fGC response observed in October (Fig. 4B) might be related to factors other than nutrition, such as reproductive cycles, but more data collection is needed to test these theories.

We assessed the influence of eight different temporal scales (between disturbance and fecal collection) of vessel counts on gray whale fGC concentrations and determined that vessel count on the day prior to sample collection best explained fGC data. Thus, we hypothesize that gut transit time of stress hormones in gray whales may occur within ~ 24 hours of the disturbance event. We recognize that whales likely moved location within the 24 hours prior to fecal sample collection, but these whales were not tagged, so we are unable to quantify the exact number of vessels encountered or the associated sound exposure level of individual whales in the days prior to sample collection. However, our non-invasive photo-identification analysis indicates that most whales have high residency time in the study region and therefore were likely exposed to the vessel traffic and measured noise or very similar levels encountered in the study area. Our approach to summarize and assess vessel traffic and possible associated noise levels across the entire study area on each day assumes all whales within the study area endure a common vessel and noise exposure level each day regardless of location. Yet, unaccounted for whale movement across the study area may obscure finer-scale relationships between variation in local vessel abundance and traffic and later whale excretion of GCs, potentially reducing correlations for longer time lags. However, our results align with previous studies indicating that large mammals generally have gut transit times between 12 hours and 4 days, with a ~ 24-hour lag common in large carnivores^31,41,44^.

A complete understanding of the impact of vessel traffic and its associated noise on whale physiology remains unclear, and other factors unaccounted for in this study may influence gray whale GC concentrations, such as contaminants^56^, predation events^57^, and infections^58^. Vessel traffic can affect whale physiology via multiple pathways, including behavioral disturbance and altered metabolic rates caused by increased risk of collision with vessels^21,29^, elevated energetic expenditure to call more loudly or missed acoustic signals from conspecifics and predators due to masking effects, or a combination of these and other possibilities. This population of gray whales does show signs of propeller strike^41^ and is also subject to whale watch activities with documented behavioral disturbance from vessel traffic within our study region^15^. Thus, disentangling the impacts of vessel traffic from other impacts will require further assessment and larger sample sizes, as well as analyzing other confounding variables in parallel.

In addition to a large and active sportfishing community, Newport and Depoe Bay harbors also support many large commercial fishing vessels and approximately five whale watch operations. The Newport channel is also dredged by a large U.S. Army Corps of Engineers vessel during summer months and the harbor is home to the NOAA Pacific Fleet of large research vessels. Unfortunately, these vessel activities were not available in the vessel count data from Oregon Department of Fish and Wildlife that focuses on the sportfishing effort from these ports. This unaccounted-for vessel traffic may partly explain the variation in data points around the trend lines between vessel counts and daily ambient sound levels (Fig. 2), and the unexplained variance in the linear model.

Anthropogenic disturbance, including vessel traffic, can be stressful for marine mammals^30,31^, and often correlates with deterioration in overall health^55,59,60^, depressed immune systems, and increased mortality^61^. Despite our small sample size, we demonstrate correlations between vessel counts and whale stress-related hormone concentrations at an individual level and within short temporal periods (~ 24 hours). Furthermore, we provide initial evidence on a potential association between ocean noise originated from vessel traffic and fGC in gray whales. Our gray whale case study results can inform how other, less accessible baleen whale populations may physiologically respond to disturbance. However, it is important to note that our results are preliminary and not fully conclusive. A larger sample size is needed to confirm and further unravel patterns. Future work should build upon these documented associations between vessel traffic and stress responses in baleen whales to assess impacts on individual vital rates and subsequent population dynamics. Furthermore, future studies should directly explore potential correlations between stress responses in baleen whales and ocean noise. With continued assessment of physiological impacts of vessel disturbance and resulting ocean noise on baleen whales, environmental managers can develop effective regulations, such as determining thresholds for the number, speed and proximity of vessels to reduce sound levels near whale habitats and mitigate impacts on vulnerable and protected whale populations.

## Methods

### Gray whale data

All methods were carried out in accordance with relevant guidelines and regulations. This project was approved by the Oregon State University Institutional Animal Care and Use Committee (IACUC-2019-0008) and complies with the ARRIVE guidelines. All gray whale data collection was carried out under a research permit from NOAA/NMFS (#16011 and #21678, issued to John Calambokidis).

We used a small research vessel (5.4 m rigid-hulled inflatable boat) to collect gray whale data over the course of three foraging seasons (May to October from 2016 to 2018) along the central coast of Oregon, USA, including near the ports of Newport and Depoe Bay (Fig. 1). Gray whale visual surveys did not follow a standardized transect line, but rather prioritized maximizing whale encounters to collect individual-based data. Whale photographs were taken for individual photo-identification and drone-based videos were recorded for photogrammetry analysis when weather conditions and whale behavior were suitable (methods are fully described in^62^). We also opportunistically collected fecal samples at the whale sightings using two 300 µm nylon mesh dipnets (methods are fully described in^41^). Samples were transferred to sterile plastic jars and placed on ice until stored in a freezer (− 20 °C) for later analysis. Date, time, and location were documented for each fecal sample, as well as the matching photo for the specific individual.

We used Adobe Bridge software (version 8.0.1.282) for photo-identification analysis. Photographs were compared to long-term gray whale catalogs held by the Marine Mammal Institute at Oregon State University and Cascadia Research Collective (Olympia, WA, USA) to obtain individual sex and minimum age information based on date of first sighting. If sex was unknown, sex was determined through fecal genetic analyses (methods are fully described in^55^). Given the unknown movements of our study whales prior to fecal sample collection, we assessed the residency time (in days) of these whales within the study area to justify our assumption that the whales were exposed to the measured soundscape levels and vessel counts. Through photo-identification comparison, we summed the number of days a sampled whale was in the study area (1) prior to sample collection in that year, and (2) in total that year.

Images of whales flat and straight at the surface were extracted from drone video recordings using VLC software (version 2.2.8) and scored as good or poor quality based on pre-defined attributes^55^. Only images scored as good were measured in custom MATLAB (version 9.3.0.7, release 2017b) software, producing a series of ten morphometric attributes that describe the whale’s body condition. These metrics were assessed in R (version 3.5.0^63^) to calculate a final metric called Body Area Index (BAI), which is a unitless and length-standardized metric of body condition that allows comparisons among individuals of different lengths and demographic units (e.g., calves and adults, or males and females^55^). We applied a coefficient of variance threshold of 5% for both whale length and BAI measurements to improve accuracy. Whales were assigned to a demographic unit based on sex and maturity status in each year^55^ based on fieldwork observations, photo-identification, and photogrammetry results. The BAI metric has been successfully implemented to document variation in body condition in this specific gray whale population across foraging seasons^55^.

Fecal samples were filtered, desalted, and freeze-dried^41^. Dried, processed samples were mixed and weighed to the nearest 0.2 g; samples below 0.02 g were excluded from the analysis to avoid inflated values ["small sample effect"; see ^33,43^]. Fecal hormone metabolites were extracted^41^ and quantified using commercial Enzyme-linked Immunosorbent Assay kits for cortisol (#ADI-900-071), progesterone (#ADI-900-011), and testosterone (#ADI-900-065) from Enzo Life Sciences, following the manufacturer's protocols (https://www.enzolifesciences.com). Since fecal samples reflect the metabolized products of the parent hormones, the cortisol kit quantifies fGC, the progesterone kit quantifies progestin metabolites (fP), and the testosterone kit quantifies androgen metabolites (fA).

Samples were run in duplicate in 2016–2017, and in triplicate in 2018. All samples were analyzed within 11 months of collection. Our quality assurance and quality control protocols include full standard curves in each assay, re-run of any sample with > 15% coefficient of variation (CV) between replicates, and re-run of any sample outside the percent-bound range of 15–85%. Samples not conforming to these standards were analyzed again until suitable values were obtained. Values < LOD were excluded from the analysis^64^. When multiple fecal samples were collected from the same individual in the same day, we applied the values from the sample with higher mass^30^. Gray whale fecal hormone assays have been validated for all hormones described in this study, and results exhibited excellent parallelism and accuracy as well as good match to known physiological state (age, sex, reproductive state)^41^.

### Vessel traffic data

Daily counts of unique vessels from the ports of Newport and Depoe Bay during our three field seasons were obtained from the Oregon Department of Fish and Wildlife (ODFW) through video analysis at the ports. These daily vessel counts consist only of recreational craft, including commercial charters on fishing and crabbing trips, and private boats (e.g., private fishing trips, kayaks, row boats, and jet skis). Therefore, vessel activity for other purposes (e.g., whale watching, research, funerals, maintenance trips, commercial fishing, Coast Guard, and dredging) are not tracked by these counts and thus, are not accounted for in this analysis. However, during the time period of this study, no major seismic, sonar or marine construction occurred in our study area, and recreational vessel traffic in coastal areas has been found to correlate strongly with ambient noise levels^9,54^.

### Acoustic data

Concurrent with gray whale data collection, acoustic data were recorded off the coast of Newport, Oregon, from 15 June to 8 October of 2017 and 5 June to 1 October of 2018 (no acoustic data is available from 2016). A passive acoustic monitoring (PAM) hydrophone system was deployed outside the Newport harbor entrance at 44.5932 N, −124.1029 W, in 20 m water depth, and 1.25 km from the coastline (Fig. 1). The custom PAM system consisted of an omni-directional hydrophone (International Transducer Corporation transducer model ITC1032) with sensitivity − 192 dB re μPa V^−1^ @ 1 m combined with a low-power 16-bit data acquisition system and preamplifier housed in a fiberglass composite pressure housing^65^. The PAM system was mounted on a weighted, semi-trawl protected aluminum frame 0.5 m above the seafloor with no sea surface expression. Data were recorded at 32 kHz sample rate on a 20% duty cycle (12 min of every hour). A low frequency cutoff was applied to avoid aliasing around the Nyquist frequency, resulting in acoustic measurements that included energy up to 13 kHz. Data analysis followed previously described methods^65^. Root mean square sound pressure levels (SPL_rms_) were calculated from 50 to 1000 Hz frequency band. This frequency range captures low frequency vessel-generated noise < 1000 Hz typical of commercial and recreational boats using the ports of Newport and Depoe Bay^66^, and the sound energy from wind-related processes down to 400 Hz^67^ while avoiding the increasing surface wind generated noise that scales with wind speed and frequency up to 10,000 Hz^68^. This range is also relevant for the acoustic sensitivity of gray whales and overlaps with two of the most frequent call types observed in the northeastern Pacific (known as “M1 and M3 calls”)^69–71^. Although audiograms for gray whales are not available, since they produce calls in this range it is likely that they are able to hear at, or are acoustically sensitive in the frequencies of the calls they produce.

A daily median SPL_rms_ (50–1000 Hz) calculated from 6 a.m. to 7 p.m. Pacific Daylight Time provides a measure of the 50^th^ percentile, or typical sound levels, associated with vessel activity at the harbor entrance during the busiest daytime hours of each day.

### Wind speed data

Wind-generated surface noise also contributes to ocean soundscapes^68^. Therefore, we compiled local wind speed data during our study periods to assess and compare the contributions of wind and vessel traffic to recorded underwater ambient noise levels. Hourly wind speed data from an anemometer station located near the hydrophone on the South Beach jetty entrance to the port of Newport (station NWPO3, Newport, OR, −44.613 N, 124.067 W; Fig. 1) during our three field seasons were obtained from the NOAA National Data Buoy Center (NDBC). Times were converted to local Pacific Daylight Time. Hourly median wind speed (m/s) and a daily median wind speed value (m/s) from 6 a.m. to 7 p.m. were calculated to match noise level measurements from the deployed hydrophones.

### Data analysis

Our goal was to assess if and how gray whale fGC concentrations vary relative to vessel counts, while simultaneously accounting for the effects of months, years, demographic units, other hormone metabolites, and body condition (BAI). Therefore, every fecal sample was matched with the BAI measurement of that individual from the same day or within ± 14 days of the fecal sample collection (no change in body condition within this window was detected; paired t-test using all BAI values of individuals assessed within 14 days in 2016, 2017 and 2018: n = 61, *p* = 0.86, df = 60, t =  − 0.174).

Only mature males and non-pregnant, non-lactating mature females were included in this analysis to minimize the known impact of normal variation in fGC concentrations due to life history phases^33,41,72^. It was assumed that adult females swimming in close association with a calf were lactating females and that they were pregnant in the previous year, based on a gestation of 13 months^73^.

All statistical analyses were conducted in R software^63^ with a significance level of 0.05. Normality of all variables was tested using the Shapiro–Wilk normality test, with non-normal variables log-transformed (log-normal [value + 1]) before further analysis.

Linear regressions were performed using the *lm* function in R to test for correlations between (1) daily vessel count data from both ports and ocean noise (daily median SPL_rms_) in 2017 and 2018, and (2) ocean noise in 2017 and 2018 and daily median wind speed. To further explore the temporal patterns in underwater sound levels and any potential correlation with local wind patterns, the median noise levels for each hour of each day recorded by the Newport hydrophone (50–1000 Hz) in 2017 and 2018 were plotted in MATLAB (version 9.7.0.1190202–R2019b) alongside the hourly median wind speed over the same time period (Fig. 3).

Linear mixed models (LMM) were conducted using the *lme4* package in R^74^, to assess the effects of vessel counts, month, year, sex, BAI, and other hormone metabolites on fGC concentrations. The ports of Newport and Depoe Bay are 22 km apart, which is within the daily travel range of a gray whale (*L. Lemos, pers. obs.*; based on field observations and photo-identification analysis). As daily vessel counts at Newport and Depoe Bay were positively correlated (rate of change = 3.176, F_1,233_ = 520.6, R^2^ = 0.689, *p* < 0.001), we assumed that vessel activity from both ports influences acoustic conditions within the study area. Therefore, we summed vessel counts from the two ports for analysis relative to fGC concentrations.

Due to uncertainty regarding gut transit time (which cannot be determined experimentally in mysticetes^44^), different time lags between vessel count and fecal collection were assessed in the LMMs, including the sum of vessel counts on the same day as fecal sample collection, and on the previous 1–7 days (based on known gut transit times in large mammals of ~ 12 hours to 4 days^43,75,76^). An additional null model (model 0; Table 1), which did not included vessel counts, was run to verify the effect of vessel traffic on gray whale fGC concentration. All models included whale identification as random effect to account for pseudoreplication. Model selection was based on the lowest Akaike’s information criterion (AIC^77^). It is important to highlight that most of the models (i.e., 1-4, 7-8, b-j, l-m, o, and q) displayed a singular fit message, which may indicate overfitting of the models. Despite limiting models to just one random effect (i.e., whale ID) and reducing the number of random effects included in the model, singular fits persisted. Since this warning message may also relate to the variance of one or more combinations of the effects being close to zero^78^, we believe that the singular fit is due to the low sample size while splitting the data between many relevant fixed and random effects. Therefore, exclusion of relevant variables may not necessarily resolve the issue; thus, we selected models based on AIC, parsimony, and inclusion of variables relevant for explaining fGC variation (Table 1). After the most influential temporal scale for vessel counts was determined, additional LMMs were run with varied combinations of the fixed effects. Model fit was evaluated by assessing the marginal R^2^ (R^2^m: variance explained by fixed effects) and the conditional R^2^ (R^2^c: variance explained by both fixed and random effects) using the *MuMIn* package in R^79,80^. F-statistics and *p*-values were obtained using the *lmerTest* package^81^. To assess the relationships between vessel counts and ambient noise levels from the previous day of fecal sample collection, we conducted additional ANOVAs between these variables and months. For these posthoc analyses, he had a smaller sample size based on only 2017 and 2018 data because no hydrophone data were available for 2016.

The code and associated data to run the LMMs, linear regressions and ANOVAs are deposited in the FigShare Digital Repository: 10.6084/m9.figshare.21171886^82^.

## Supplementary Information

Supplementary Information.
