# Supplementary material for: Effects of vessel traffic and ocean noise on gray whale stress hormones
Source: Sci Rep. 2022 Nov 3;12:18580. doi: 10.1038/s41598-022-14510-5 (PMC9633705; doi:10.1038/s41598-022-14510-5)
Supplement: Supplementary file 1 — Supplementary Information. [file 41598_2022_14510_MOESM1_ESM.docx]

**Effects of vessel traffic and ocean noise on gray whale stress hormones**

**Leila S. Lemos^1,2*^, Joseph H. Haxel^3,4^, Amy Olsen^5^, Jonathan D. Burnett^6^, Angela Smith^5^, Todd E. Chandler^1^, Sharon L. Nieukirk^3^, Shawn E. Larson^5^, Kathleen E. Hunt^7^, Leigh G. Torres^1^**

^1^ Geospatial Ecology of Marine Megafauna Lab, Marine Mammal Institute, Department of Fisheries, Wildlife, and Conservation Science, Oregon State University, 2030 SE Marine Science Dr, Newport, OR, 97365, USA

^2^ Institute of Environment, College of Arts, Science & Education, Florida International University, 3000 NE 151^st^ St, North Miami, FL, 33181

^3^ Pacific Northwest National Laboratory, 1529 W Sequim Bay Rd, Sequim, WA, 98382, USA

^4^ Cooperative Institute for Marine Resources Studies, Oregon State University, 2030 SE Marine Science Dr, Newport, OR, 97365, USA

^5^ Conservation Programs and Partnerships, Seattle Aquarium, 1483 Alaskan Way Pier 59, Seattle, WA, 98101, USA

^6^ Aerial Information Systems Laboratory, Forest Engineering, Resources and Management Department, Oregon State University, Oregon, USA

^7^ Smithsonian-Mason School of Conservation & Department of Biology, George Mason University, Virginia, U.S.A.

^*^ **Correspondence**: leslemos@hotmail.com

**Supplementary Material**

**Appendix S1**

Table S1: Gray whale individuals’ sampling date, identification (ID) name, minimum age, maturity and sex.

| **Sampling date**  **(mm/dd/yyyy)** | **Whale ID** | **Minimum age** | **Maturity and sex** |
| --- | --- | --- | --- |
| 06/09/2016 | Stamp | 20 | Mature male |
| 06/12/2016 | Stamp | 20 | Mature male |
| 06/12/2016 | Bit | 16 | Mature female |
| 06/12/2016 | Knife | 21 | Mature female |
| 06/16/2016 | Knife | 21 | Mature female |
| 06/18/2016 | Knife | 21 | Mature female |
| 06/24/2016 | Stamp | 20 | Mature male |
| 06/24/2016 | Knife | 21 | Mature female |
| 06/24/2016 | Spray | 15 | Mature female |
| 08/19/2016 | Spray | 15 | Mature female |
| 09/03/2016 | Whitetip | 30 | Mature female |
| 09/07/2016 | Boomerang | 14 | Mature male |
| 09/14/2016 | Missing Pieces | 20 | Mature female |
| 07/13/2017 | Rat | 17 | Mature male |
| 07/19/2017 | Rat | 17 | Mature male |
| 08/09/2017 | Sole | 18 | Mature female |
| 08/23/2017 | Cyclone | 13 | Mature male |
| 09/15/2017 | Scarback | 21 | Mature female |
| 09/16/2017 | Clouds | 22 | Mature female |
| 09/16/2017 | Scarback | 21 | Mature female |
| 09/24/2017 | Sieve | 17 | Mature male |
| 09/24/2017 | Cobra | 15 | Mature male |
| 09/25/2017 | Scarback | 21 | Mature female |
| 10/04/2017 | Missing Pieces | 21 | Mature female |
| 10/04/2017 | Peak | 23 | Mature male |
| 10/04/2017 | Spray | 16 | Mature female |
| 10/05/2017 | Batman* | 23 | Mature male |
| 10/05/2017 | Clouds | 22 | Mature female |
| 10/05/2017 | White Hole | 17 | Mature female |
| 05/31/2018 | Sole | 19 | Mature female |
| 05/31/2018 | Slush | 10 | Mature female |
| 05/31/2018 | Peak | 24 | Mature male |
| 06/01/2018 | Orange Knuckles | 13 | Mature male |
| 06/01/2018 | Slush | 10 | Mature female |
| 06/05/2018 | Sole | 19 | Mature female |
| 06/12/2018 | Sleepy | 28 | Mature male |
| 06/12/2018 | Peak | 24 | Mature male |
| 06/12/2018 | Claw | NA | Mature female |
| 06/19/2018 | Bit | 18 | Mature female |
| 07/25/2018 | Sole | 19 | Mature female |
| 08/08/2018 | Cyclone | 14 | Mature male |
| 08/08/2018 | Scarback | 22 | Mature female |
| 08/08/2018 | Missing Pieces | 22 | Mature female |
| 08/10/2018 | Pointy | 20 | Mature male |
| 08/10/2018 | Bit | 18 | Mature female |
| 08/20/2018 | Spray | 17 | Mature female |
| 08/22/2018 | Shark | 13 | Mature female |
| 08/23/2018 | Missing Pieces | 22 | Mature female |
| 08/30/2018 | Sole | 19 | Mature female |
| 08/31/2018 | Sole | 19 | Mature female |
| 09/01/2018 | Orange Knuckles | 13 | Mature male |
| 09/01/2018 | White Hole | 18 | Mature female |
| 09/06/2018 | Scarback | 22 | Mature female |
| 09/06/2018 | Luna | 15 | Mature female |
| 09/06/2018 | Pancake | 16 | Mature female |
| 09/06/2018 | Cyclone | 14 | Mature male |
| 09/07/2018 | Moray | NA | Mature male |
| 09/07/2018 | Fish Hook | 23 | Mature male |
| 09/07/2018 | Dapper | NA | Mature male |
| 09/07/2018 | White Hole | 18 | Mature female |
| 09/14/2018 | Cyamid Marks | 16 | Mature male |
| 09/19/2018 | White Hole | 18 | Mature female |
| 09/19/2018 | Peak | 24 | Mature male |
| 09/19/2018 | Shark | 13 | Mature female |
| 09/20/2018 | Pointy | 20 | Mature male |
| 09/20/2018 | Sleepy | 28 | Mature male |
| 09/28/2018 | Peak | 24 | Mature male |


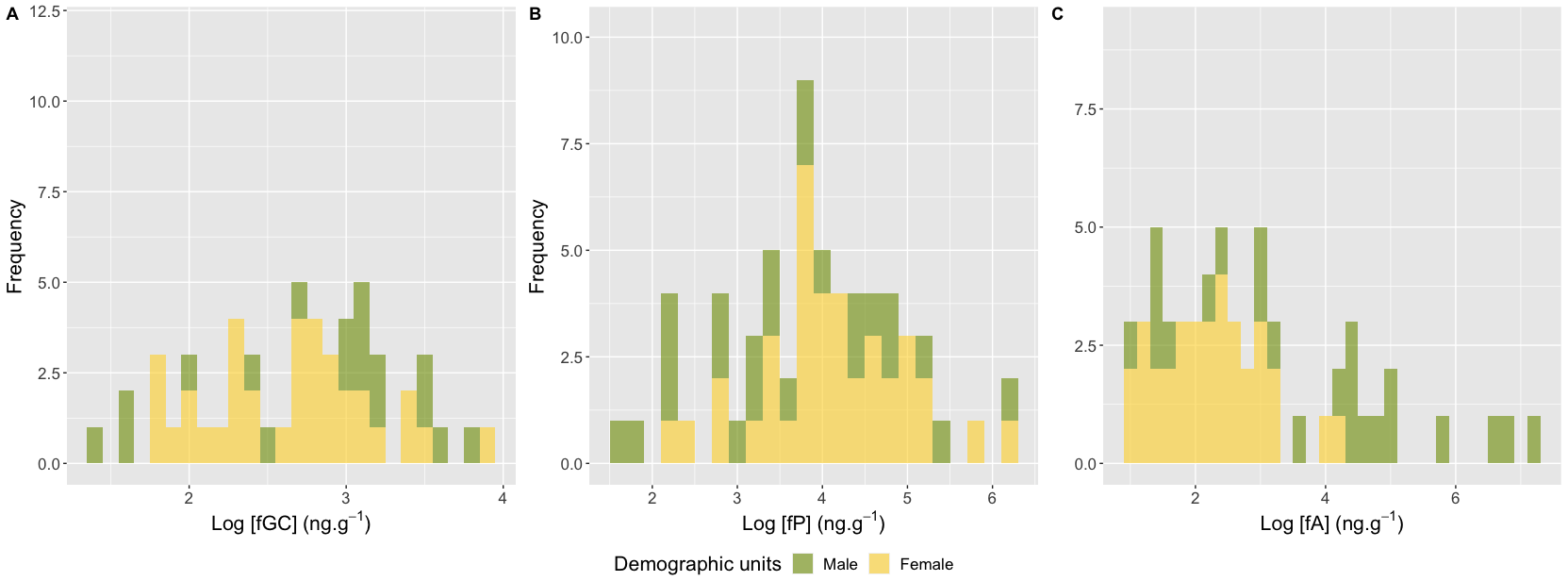


Figure S1: Frequency histograms of gray whale fecal hormone metabolite concentrations (ng.g^-1^, dried mass) by sex during June to October of 2016-2018 off the Oregon coast, USA. Individual whales may be represented multiple times in these plots as they may be re-sampled within and between years.
